# Regulatory principles governing *Salmonella* and *Yersinia* virulence

**DOI:** 10.3389/fmicb.2015.00949

**Published:** 2015-09-09

**Authors:** Marc Erhardt, Petra Dersch

**Affiliations:** ^1^Young Investigator Group Infection Biology of Salmonella, Helmholtz Centre for Infection ResearchBraunschweig, Germany; ^2^Department of Molecular Infection Biology, Helmholtz Centre for Infection ResearchBraunschweig, Germany

**Keywords:** virulence regulation, pathogenicity factors, motility, transcription factors, post-transcriptional modifications, environmental control systems regulatory RNAs, riboswitches, metabolic adaptation

## Abstract

Enteric pathogens such as *Salmonella* and *Yersinia* evolved numerous strategies to survive and proliferate in different environmental reservoirs and mammalian hosts. Deciphering common and pathogen-specific principles for how these bacteria adjust and coordinate spatiotemporal expression of virulence determinants, stress adaptation, and metabolic functions is fundamental to understand microbial pathogenesis. In order to manage sudden environmental changes, attacks by the host immune systems and microbial competition, the pathogens employ a plethora of transcriptional and post-transcriptional control elements, including transcription factors, sensory and regulatory RNAs, RNAses, and proteases, to fine-tune and control complex gene regulatory networks. Many of the contributing global regulators and the molecular mechanisms of regulation are frequently conserved between *Yersinia* and *Salmonella*. However, the interplay, arrangement, and composition of the control elements vary between these closely related enteric pathogens, which generate phenotypic differences leading to distinct pathogenic properties. In this overview we present common and different regulatory networks used by *Salmonella* and *Yersinia* to coordinate the expression of crucial motility, cell adhesion and invasion determinants, immune defense strategies, and metabolic adaptation processes. We highlight evolutionary changes of the gene regulatory circuits that result in different properties of the regulatory elements and how this influences the overall outcome of the infection process.

## Introduction

Gastrointestinal infections by pathogenic *Enterobacteriaceae* represent a serious economic and health problem worldwide. They cause severe diarrheal diseases, which are still a leading cause of death among children under five. In developed countries, the incidence of foodborne outbreaks is less problematic, but remains substantial and constitutes a significant socioeconomic burden. Globalization of food supply, introduction and persistence of the pathogens in unknown environmental niches, frequent environmental changes leading to rapid evolution of newly emerging variants, and the development of antibiotic-resistant *Enterobacteriaceae* which use the intestinal tract as main reservoir are reasons why foodborne intestinal diseases by these pathogens remain a global public health problem. The worldwide rise of antibiotic-resistant strains represents a serious threat for the treatment of gastrointestinal pathogens. Furthermore, antibiotic therapies of most gastrointestinal infections are (i) ineffective as they are unable to improve clinical symptoms or shorten duration of shedding, are (ii) skewing of the intestinal commensal community (dysbiosis) which was found to support inflammatory diseases, e.g., by enteric pathogens or other usually harmless commensals, and are (iii) associated with an increased risk of serious complications in cases such as enterohemorrhagic *Escherichia coli* (EHEC).

Many cases of gastrointestinal infections in Europa and North America are caused by members of the genus *Salmonella* and *Yersinia*. The enteric representatives of both genera are gram-negative, facultative anaerobic and motile bacteria, which are usually transmitted by infected animals, or contaminated food or water via the fecal–oral route. Enteropathogenic *Salmonella* and *Yersinia* species can occupy many different environmental habitats, persist in certain domestic and wild animal reservoir hosts (in particular cattle, swine, poultry, wild birds, pet reptiles), and are routinely isolated from ground water, soil, plants, and insects (Fredriksson-Ahomaa et al., 2006; Hoelzer et al., 2011; Fredriksson-Ahomaa, 2012; Wiedemann et al., 2014). Undercooked chicken and eggs are considered the major infection source of *salmonellae*, whereas undercooked pork, vegetables, and lettuce are responsible for most *Yersinia* infections (Hoelzer et al., 2011; Fredriksson-Ahomaa, 2012). Enteric *Salmonella* and *Yersinia* species cause various gut-associated symptoms (e.g., enteritis, ileitis, colitis, vomitting, intestinal cramping, and inflammatory diarrhea), and in rare cases they can lead to systemic infections and induce extra-intestinal sequelae such as fatal respiratory, hepatic, spleen, and/or neurological damage, erythema nodosum and reactive arthritis (Bottone, 1999; Koornhof et al., 1999; Ohl and Miller, 2001). Inflammatory diarrhea, which is typically associated with the highly invasive *Yersinia* species *Y. enterocolitica* and *Y. pseudotuberculosis*, and enteroinvasive *Salmonella* serotypes is characterized by an acutely increased vascular permeability and the recruitment of neutrophils which result in the formation of tissue exudates and necrotic lesions (Autenrieth and Firsching, 1996; Tsolis et al., 2008). Invasion of the bacteria from the intestinal tract into underlying lymphatic follicles (Peyer’s patches) can lead to the ulceration of the tissue and abdominal pain that mimics appendicitis. The detection of the invasive bacteria by the innate immune system also results in the production of pyrogenic cytokines (IL-1β, IL-6, TNF-α and interferons) that increase the host’s body temperature (typhoid/enteric fever) (Tsolis et al., 2008; Gu et al., 2013).

**FIGURE 1:**
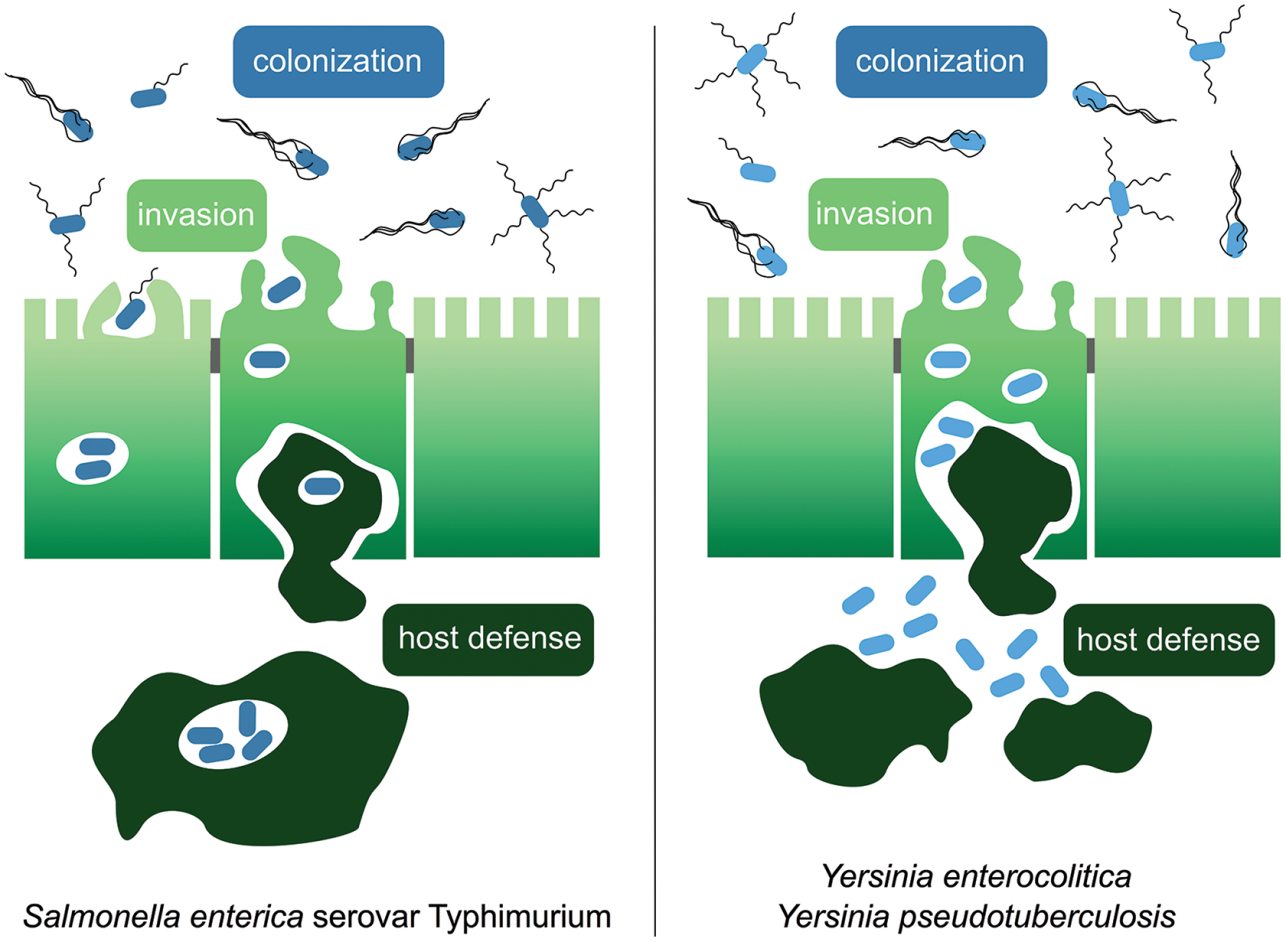
**The course of infection by enteric *Yersinia* species and *Salmonella enterica* serovar Typhimurium.** Enteropathogenic *Salmonella* Typhimurium utilizes flagellar motility for host colonization in the gut lumen and directed movement toward the epithelial layer. *Salmonella* attaches to the intestinal epithelium using a variety of adhesins and secretion of effector proteins via the Spi-1 encoded injectisome device for invasion of M cells or other enterocytes. The bacteria survive and replicate within *Salmonella*-containing vacuoles inside epithelial cells and phagocytes. The enteropathogenic *Yersinia* species *Y. enterocolitica* and *Y. pseudotuberculosis* are ingested via contaminated food and enter the lymphatic system through the M cells in the small intestine. In the lymphatic tissues they reside predominantly outside of the cells.

Enteric *Yersinia* and *Salmonella* serotypes are well adapted for survival in a variety of external environments and persistence in various host animals. The bacteria adjust rapidly to several extremely different and stressful environments during the invasion process, such as gastric acidity, increased osmolarity, changing nutrient/ion availability, and competition with the host’s microbiota in the intestine (Fabrega and Vila, 2013; Heroven and Dersch, 2014). In order to succeed in invading the host, they employ a wide variety of virulence factors, such as flagella, host colonization factors (fimbriae, adhesins/invasins), and many secreted toxins and effector proteins. These molecules are needed to propel the bacteria to target cells, attach to, infect and survive inside host cells and protect the pathogen against host immune responses (**Figure 1**). To establish a successful infection, process, these virulence factors and adequate metabolic pathways must be expressed at the correct spatiotemporal conditions. Consequently, a substantial cross-talk between different pathogenicity elements and physiological adaptation processes has to exist. The correct spatiotemporal expression of the various virulence constituents is achieved by regulation at the transcriptional, post-transcriptional and post-translational levels by a myriad of transcription factors, nucleoid-associated proteins, regulatory small RNAs (sRNAs) and RNases. Moreover, a growing set of small signaling molecules and different proteases controls the activity of enzymes and virulence-relevant factors during the infection process. The majority of the contributing regulators and control mechanisms are highly conserved between *salmonellae* and *yersiniae*, but the regulatory network composition and architecture are often changed. In the following chapters we will compare regulatory factors and global networks controlling early and late stages of the infection process of *Salmonella* and *Yersinia* to highlight conserved regulatory elements, but also changes that provided an important source for the divergence of the different genera with respect to their virulence-relevant physiological and pathogenic properties.

## Regulatory Circuits Controlling Early Stages of Host Colonization

### Survival in the Intestinal Lumen

The gastrointestinal tract of mammals is a rich source of food for enteric pathogens as it contains a large variety of simple and polymeric sugars, amino acids, peptides, and proteins, as well as lipids and other carbon- and nitrogen-containing metabolites. The availability of the nutrients varies significantly between hosts, different sections of the intestinal tract and different stages of the infection process, as it depends strongly on (i) the diet of the host, (ii) the composition and activity of the intestinal microbiota, which includes 10^14^ well-adapted bacteria from more than 400 species, (iii) absorption by the intestinal cells, and (iv) inflammation and hypoxic conditions induced by the host immune response during the progress of an infection (Nizet and Johnson, 2009; Rohmer et al., 2011). Moreover, the host limits the pathogen’s access to essential ions, including magnesium, iron, and zinc (Brown et al., 2008; Abu Kwaik and Bumann, 2013; Zhang and Rubin, 2013). Consequently, *Salmonella* and *Yersinia* must either grow on non-utilized energy sources or metabolic end products, or metabolize nutrients and import ions much more efficiently to ensure maximal fitness and competiveness against the intestinal microbiota.

An important common characteristic of enteropathogenic *Salmonella* and *Yersinia* strains is that they possess a highly complex metabolism with many redundant or alternative catabolic and biosynthetic pathways. This allows them to utilize a large variety of organic energy sources and renders them very flexible and robust against sudden changes of nutrient availability. Another basis for their success is their ability to select the best energy sources among provided nutrients to gain the most benefit for lowest costs to optimize their biological fitness (Dandekar et al., 2014; Heroven and Dersch, 2014). Furthermore, they possess a plethora of sophisticated sensing and signal transduction strategies to adapt to variation in the nutrient composition during the course of an infection. Moreover, a large number of conserved regulatory proteins has been identified over the past years, which are implicated in metabolic control (**Table 1**), and a steadily increasing number of post-transcriptional control systems, including non-coding RNAs, and small signaling molecules such as ppGpp, cAMP, and c-di-GMP has been identified as additional elements controlling virulence and metabolism.

**Table 1:** Comparison of the protein homology of major regulatory factors of *Salmonella enterica* serovar Typhimurium (ST) and *Yersinia pseudotuberculosis* (Ypt) using Basic Local Alignment Search Tool (BLAST) analysis.

Gene name ST strain LT2	Locus tag ST strain LT2	Gene name Ypt strain YPIII	Locus tag Ypt strain YPIII	Sequence coverage (%)	Amino acids identity (%)	Mode of regulation	Virulence pathway
BarA	STM2958	BarA	YPK_3451	99	59	TCS	Colonization, invasion, host defense
ClpP	STM0448	ClpP	YPK_3234	100	89	Protease	Colonization, invasion
CpxA/CpxS	STM4058	CpxA	YPK_4133	99	81	TCS	Host defense
CpxR	STM4059	CpxR	YPK_4132	100	89	TCS	Host defense
Crp	STM3466	Crp	YPK_0248	100	99	Transcription	Colonization, invasion, host defense
CsrA	STM2826	CsrA	YPK_3372	100	95	Translation	Colonization, invasion, host defense
Dam	STM3484	dam	YPK_0228	96	70	Translation	Invasion
DnaK	STM0012	DnaK	YPK_3594	100	92	Protein stability	Colonization
EnvZ	STM3501	EnvZ	YPK_0173	97	88	TCS	Colonization, invasion, host defense
FimZ	STM0549		*YPK_2269*	98	48	Transcription	Colonization
FimZ	STM0549		*YPK_2499*	98	31		
Fis	STM3385	Fis	YPK_0452	100	98	Transcription	Colonization, invasion, host defense
FlhC	STM1924.S	FlhC	YPK_1746	100	82	Transcription	Colonization
FlhD	STM1925	FlhD	YPK_1745	97	76	Transcription	Colonization
FliA	STM1956	FliA	YPK_2380	99	83	Transcription	Colonization, invasion
FliT	STM1962	FliT	YPK_2384	85	36	Protein stability	Colonization
FliZ	STM1955	FliZ	YPK_2378	91	55	Transcription, translation	Colonization, invasion
H-NS	STM1751	H-NS	YPK_2074	98	87	Transcription	Colonization, invasion, host defense
HdfR/ YifA	STM3897		*YPK_4064*	98	62	Transcription	Colonization
Hha	STM0473	YmoA	YPK_3214	93	82	Transcription	Colonization, invasion, host defense
HilA	STM2876		NA	NA	NA	Transcription	Invasion
HilC/ SirC	STM2867		NA	NA	NA	Transcription	Invasion
HilD	STM2875		NA	NA	NA	Transcription	Colonization, invasion, host defense
HilE	STM4509.S		*YPK_0803*	83	30	Protein activity	Invasion
IhfA	STM1339		YPK_1826	98	94	Transcription	Host defense
InvF	STM2899		NA	NA	NA	Transcription	Invasion
IscR/ YfhP	STM2544	IscR	YPK_1275	88	79	Transcription	Host defense
Lon	STM0450	Lon	YPK_3232	100	91	Protease	Colonization, invasion
LrhA	STM2330	RovM	YPK_1559	92	72	Transcription	Colonization, invasion
NA	NA	LcrQ/YscM	pYV0089	NA	NA	Translation	Host defense
NA	NA	YopD	pYV0054	NA	NA	Translation	Host defense
OmpR	STM3502	OmpR	YPK_0172	100	99	TCS	Colonization, invasion, host defense
PhoB	STM0397	PhoB	YPK_3276	100	90	TCS	Colonization, invasion
PhoP	STM1231	PhoP	YPK_1715	99	79	TCS	Invasion, host defense
PhoQ	STM1230	PhoQ	YPK_1714	96	62	TCS	Invasion, host defense
PhoR	STM0398	PhoR	YPK_3275	99	72	TCS	Colonization, invasion
QseB/ YgiX	STM3177		*YPK_3741*	98	48	TCS	Colonization
QseC/ YgiY	STM3178		*YPK_3742*	61	34	TCS	Colonization
RcsB	STM2270	RcsB	YPK_2843	100	92	TCS	Colonization, invasion, host defense
RcsD/ YojN	STM2269	RcsD	YPK_2842	98	47	TCS	Colonization, invasion, host defense
RflM/EcnR	STM4337	RcsA	YPK_1671	93	29	Transcription	Colonization
RhaS	STM4048	LcrF	pYV0076	96	22	Transcription	Host defense
Rnase E/rne	STM1185	Rnase E	YPK_1677	100	64	Translation	Invasion, host defense
RscC	STM2271	RscC	YPK_2844	99	58	TCS	Colonization, invasion, host defense
RtsA	STM4315		NA	NA	NA	Transcription	Invasion
RtsB	STM4314		*YPK_3033*	62	41	Transcription	Colonization, invasion
RtsB	STM4314		*YPK_1465*	56	35		
SdiA	STM1950	YpsR	YPK_1655	91	29	Transcription	Colonization
SdiA	STM1950	YtbR	YPK_0791	81	28	Transcription	Colonization
SirA	STM1947	SirA	YPK_2356	99	84	TCS	Colonization, invasion, host defense
SlyA	STM1444	RovA	YPK_1876	98	75	Transcription	Colonization, invasion, host defense
SpiR	STM1392		*YPK_3918*	98	44	TCS	Host defense
SscA	STM1399	LcrH	pYV0056	83	27	Translation	Host defense
SsrB	STM1391	SsrB	YPK_3919	96	55	TCS	Host defense
YdiV	STM1344		*YPK_1543*	91	22	Protein stability	Colonization, invasion
YhjH	STM3611		*YPK_0103*	91	49	Protein activity	Colonization

*Sequence coverage reports the length of the identified homologous protein sequence with the indicated amino acid identity. In the majority, the true orthologs of the query protein sequences are listed, whereas close homologs with unknown regulatory function and low sequence identity are given in italics. NA = not available.*

#### Sensing Intestinal Stimuli by Two-Component Regulatory Systems

**FIGURE 2:**
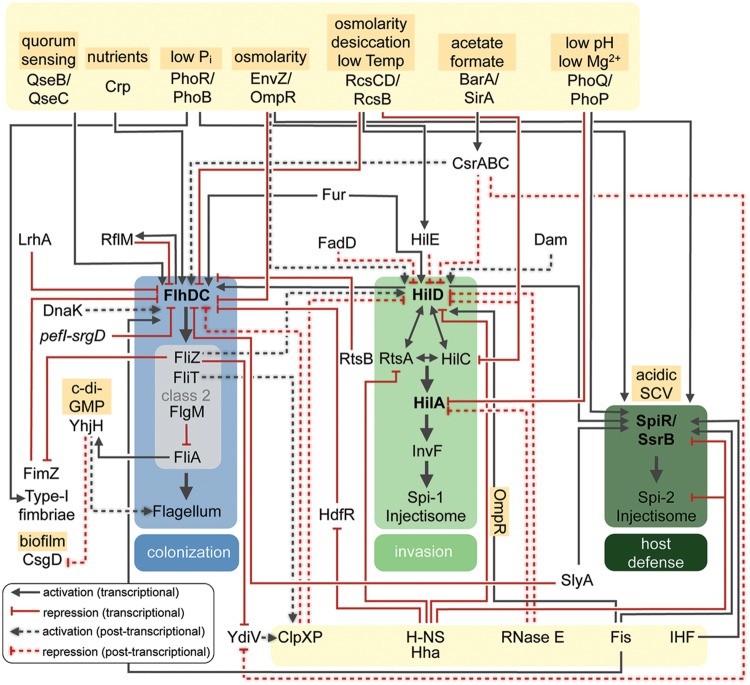
**Regulatory networks controlling *Salmonella* virulence factors.** Overview of regulatory and environmental factors that control expression of the major pathogenicity traits of *Salmonella* needed for host colonization, invasion and host defense. Transcriptional or post-transcriptional regulatory effects are listed.

**FIGURE 3:**
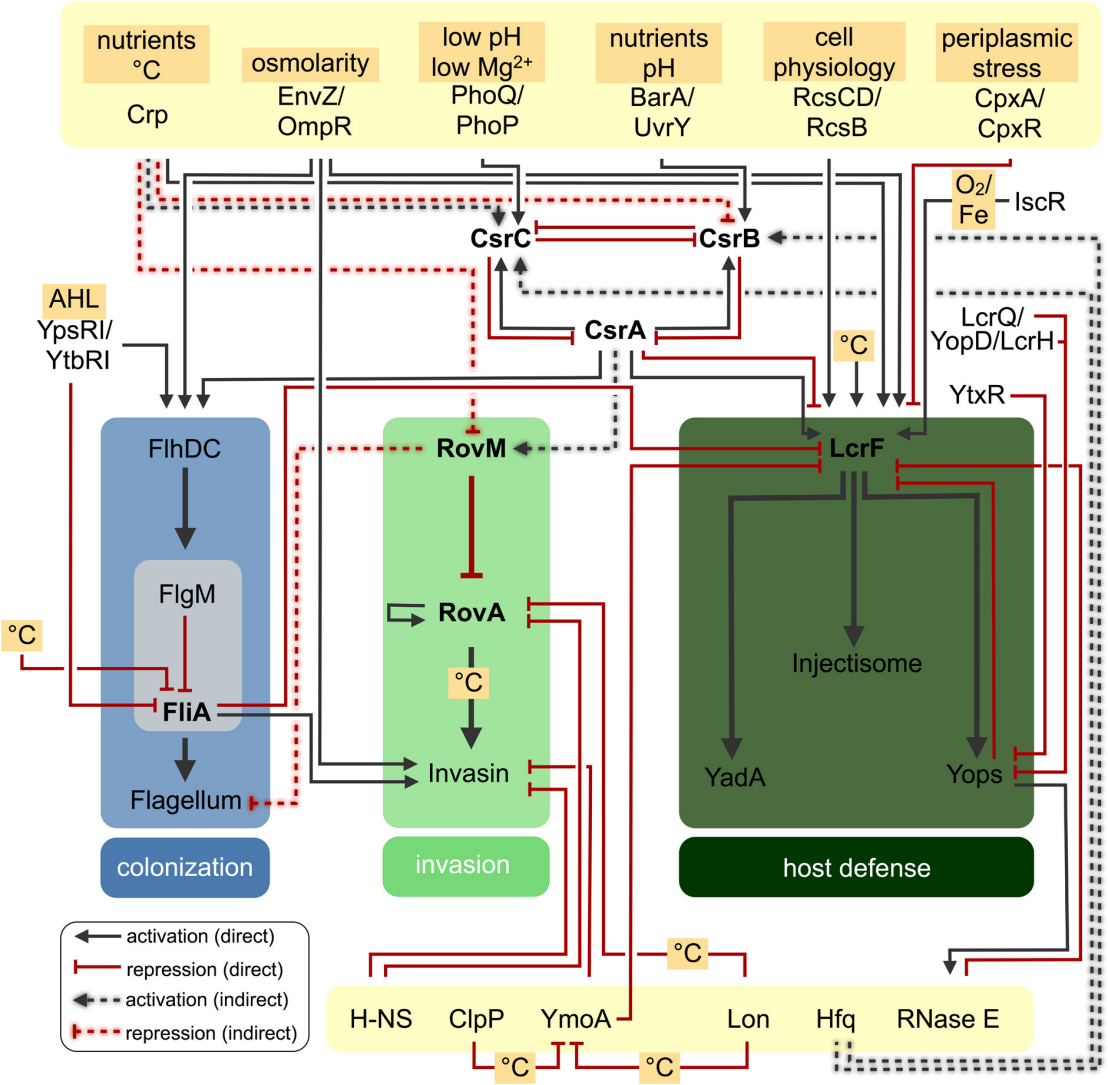
**Regulatory networks controlling *Yersinia* virulence factors.** Regulatory networks controlling expression of motility, adhesion and injectisome virulence traits in *Yersinia*. Direct or indirect regulatory effects of various factors are indicated.

External stimuli, which are relevant for metabolic adaptation and virulence are frequently sensed by bacterial two component systems (TCSs) and converted into an adaptive cellular response. Among the most virulence-relevant TCS are the pleiotropic PhoP/PhoQ, EnvZ/OmpR, and BarA/SirA(UvrY) systems (Groisman, 2001; Groisman and Mouslim, 2006) (**Figures 2** and **3**). They are composed of the membrane-bound sensor kinases PhoQ, EnvZ, and BarA that sense the environmental signals and phosphorylate the cytoplasmic response regulator PhoP, OmpR, and UvrY(SirA). The TCS PhoP/PhoQ responds to low magnesium, low pH environments, and host-secreted cationic antimicrobial peptides (Groisman, 2001). The global regulator PhoP controls a very complex network of genes, whereby the individual *Yersinia* and *Salmonella* PhoP regulons have considerable differences: (i) in the molecular architecture of the regulatory sequences and promoters, and (ii) in amino acid alterations in the conserved PhoP regulator itself. This enables both PhoP response regulators to retain the ability to transcribe the core members of the regulon in both pathogens and also allows inclusion of newly acquired genes into the ancestral regulatory circuit (Perez and Groisman, 2009). In *Salmonella*, the PhoP/PhoQ system is essential for virulence and survival within macrophages (Miller et al., 1989). The response regulator PhoP represses *hilA* and the *prg* (PhoP-repressed genes) genes (Pegues et al., 1995; Bajaj et al., 1996), whereas transcription of PhoP-activated genes (*pag*) required for survival within macrophages is activated (Miller et al., 1989; Belden and Miller, 1994). PhoP also controls expression of *Salmonella* pathogenicity island-2 (Spi-2) by binding to the *ssrB* promoter region and the 5′-UTR of the *spiR* transcript (Belden and Miller, 1994). In contrast, in *yersiniae* the PhoP/PhoQ system has been found to promote proliferation of human pathogenic *yersiniae* within professional phagocytes *in vitro*, but its role during pathogenesis is less defined (Grabenstein et al., 2004, 2006; Flamez et al., 2007). Recent studies indicate that strain-specific differences that remodel expression of PhoP-dependent control functions appear to influence the overall outcome of the virulence phenotype (Grabenstein et al., 2004; Bozue et al., 2011; Nuss et al., 2014; Pisano et al., 2014). The PhoP/PhoQ system of *Yersinia* was shown to control modification of lipid A linked to antimicrobial peptide resistance and promotes survival and proliferation in macrophages and neutrophils (Grabenstein et al., 2004; Reines et al., 2012). Another important virulence control system, which is under PhoP/PhoQ control, is the carbon storage regulator (Csr) system coordinating the expression of important *Yersinia* adhesion factors (e.g., invasin), motility and multiple virulence-relevant metabolic pathways (see also Conclusion and Outlook; Heroven et al., 2008; Bücker et al., 2014; Nuss et al., 2014).

The TCS OmpR/EnvZ was originally identified as regulator system controlling the outer membrane porins in response to osmolarity and temperature, but later it was found that it also plays an essential role in controlling the expression of *Yersinia* and *Salmonella* virulence functions (Dorman et al., 1989; Dorrell et al., 1998; Lee et al., 2000; Brzostek et al., 2012). In *Yersinia*, it is required for serum resistance, survival within macrophages, biofilm formation, and it influences the adhesion/invasion abilities (e.g., invasin, Ail). It was further shown to control expression of the AcrAB-TolC multidrug eﬄux pump, urease, and a type-VI secretion system (T6SS-4) (Raczkowska et al., 2011a, 2015; Brzostek et al., 2012; Reines et al., 2012; Gueguen et al., 2013; Skorek et al., 2013; Zhang et al., 2013). Recently, it was also demonstrated that OmpR of *Yersinia* is involved in the control of motility and flagellation by activation of the flagellar operon *flhDC* (Hu et al., 2009; Raczkowska et al., 2011b). In *Salmonella*, the EnvZ-OmpR TCS also regulates flagellar and virulence genes, but in this pathogen OmpR represses *flhDC* in response to extracellular osmolarity. It has been further implicated in post-transcriptional regulation of the Spi-1-encoded regulator HilD (Ellermeier et al., 2005; Golubeva et al., 2012) and functions as an activator of Spi-2 virulence genes (Lee et al., 2000; see also Regulation of Motility, Attachment and Invasion of the Intestinal Epithelium).

Another virulence-relevant TCS, the BarA/UvrY (SirA) system, is activated by metabolic end products (e.g., short-chain fatty acids such as format, acetate) and an imbalance of the TCA cycle (Takeuchi et al., 2009; Chavez et al., 2010). This signal transduction system is conserved in many γ-proteobacteria and controls the expression of small regulatory RNAs with unpaired GGA motifs, which are part of the Csr system (Heeb and Haas, 2001; Lapouge et al., 2007). In *Yersinia* and *Salmonella* the BarA/UvrY(SirA) system controls the expression of cell invasion genes. It is required for the expression of the *Salmonella* Spi-1 invasion genes, and transcription of these genes was shown to be restored in a *barA* mutant by the addition of acetate, but not with butyrate. This indicated that the concentration and composition of short-chain fatty acids in the distal ileum provides a signal for productive infection by *Salmonella* (Altier et al., 2000; Lawhon et al., 2002). SirA(UvrY) indirectly activates Spi-1 gene expression by controlling expression and function of Csr-type regulatory RNAs (CsrB, CsrC). CsrB and CsrC inhibit the function of the RNA-binding protein CsrA, which in turn prevents translation of *hilD*, a major activator of Spi-1 (see also Attachment and Invasion of the Intestinal Epithelium, **Figure 2**; Altier et al., 2000; Teplitski et al., 2006; Martinez et al., 2011). Similar to *Salmonella*, BarA/UvrY(SirA) controls expression of the CsrB RNA of *Yersinia*, which also regulates the expression of host cell adhesion and invasion genes (e.g., invasin), however, through a very different signal transduction cascade (see Attachment and Invasion of the Intestinal Epithelium, **Figure 3**).

#### Metabolic Adaptation by Global Regulatory Systems of the Carbon Metabolism

Several global regulators, which are highly conserved between *Salmonella* and *Yersinia*, are known that govern multiple cascades of control elements and complex networks in order to manage metabolic adaptation and coordinate this process with pathogenicity mechanisms. Among them is the cAMP receptor protein Crp. This regulator helps the pathogens to rank available C-sources in order to optimize their metabolism (Gorke and Stulke, 2008; Poncet et al., 2009). Primarily, the bacteria check for the availability of readily digestible sugars such as glucose and fructose. In the absence of these efficiently utilizable sugars, the signal metabolite cAMP is produced by the adenylate cyclase, which then binds to Crp to form an active cAMP-Crp complex (Hanamura and Aiba, 1991; Ishizuka et al., 1994). This metabolic sensor complex was recently shown to control more than 6% of the genes in *Y. pseudotuberculosis* (Heroven et al., 2012b; Nuss et al., 2015). *crp* mutants of *Y. pseudotuberculosis* and *Y. enterocolitica* are strongly attenuated, and comparative transcriptomics, metabolomics, and phenotypic microarrays showed that Crp is required for growth on different carbon sources and promotes survival under carbon, nitrogen, and phosphate limitation (Petersen and Young, 2002; Heroven et al., 2012b; Nuss et al., 2015). In a recent study using RNA-Seq we could also demonstrate a massive remodeling of the Crp-controlled network in response to temperature and discovered Crp as a transcriptional master regulator of numerous conserved regulatory RNAs, which adjust the *Yersinia* metabolism and fitness to the requirements of their life-style in the intestine (Nuss et al., 2015). Moreover, Crp was found to link nutrient status/carbon metabolism and the regulation of virulence factors, e.g., via the control of the switching of the two Csr-RNAs CsrB and CsrC of *Y. pseudotuberculosi*s (Heroven et al., 2012b). Similarly, Crp of *Salmonella enterica* serovar Typhimurium was shown to coregulate carbon metabolism and virulence directly and indirectly through the Csr system (see below and Section Attachment and Invasion of the Intestinal Epithelium; Teplitski et al., 2006), indicating that this regulator fulfills a similar role in this enteric pathogen. In fact, a *crp* mutant of *Salmonella* is completely attenuated in mice (Zhang et al., 1997).

As mentioned above, the other crucial global metabolic control system of *Yersinia* and *Salmonella*, the Csr system, is constituted of the global regulatory RNA-binding protein CsrA and the two antagonizing non-coding sRNAs CsrB and CsrC that bind and sequester multiple CsrA dimers. This prevents CsrA binding to lower affinity mRNA targets, which results in altered mRNA translation and/or stability (Heroven et al., 2012a; Romeo et al., 2012, 2013; Vakulskas et al., 2015). The CsrA protein interacts generally with hairpin structures located in the 5′-untranslated regions of its target genes that possess a GGA motif within the single-stranded loop with conserved flanking regions (RUACAR**GGA**) (Vakulskas et al., 2015). For instance, the binding of a CsrA dimer to one GGA-containing site was shown to allow bridging and binding to another site overlapping the ribosome-binding site of a target gene which resulted in translation repression (Mercante et al., 2009). CsrA has a global influence on the *Yersinia* transcriptome, and in total approximately 20% of the CsrA-dependent genes of *Y. pseudotuberculosis* are involved in a myriad of metabolic processes and are controlled in response to ions and availability of carbon sources (Heroven et al., 2012a; Bücker et al., 2014). Similarly, the absence of the *csrA* gene in *Salmonella* also greatly reduces mRNA levels of a large number of metabolic genes, e.g., genes for the utilization of maltose, propanediol, and ethanolamine (common carbon sources/nutrients of the intestinal tract), tetrathionate metabolism, and hydrogen sulfide production. Most importantly, these metabolic functions enable *Salmonella* to use tetrathionate as a terminal electron acceptor in the intestinal tract and allow the microbe to grow on degradation products of the microbiota (Lawhon et al., 2003; Winter et al., 2010).

### Regulation of Motility

Flagella-mediated motility is used by *Salmonella* and *Yersinia* for movement in the intestinal lumen. The chemotactic motility is required for efficient colonization in the intestinal tract, host cell invasion and induction of pathogenesis. It allows the pathogen to benefit from the increased nutrient availability in the inflamed intestine, and initiates host cell contact and invasion (Young et al., 2000; Stecher et al., 2004, 2008). Flagella are rotary motility organelles and rotation of the rigid flagellar filaments provides the pathogens with propulsion forces for chemotactic movement through liquids (swimming) and highly viscous environments or surfaces (swarming) (Chevance and Hughes, 2008). The bacterial flagellum is assembled by a flagellar-specific type-III secretion system (f-T3SS) that is highly homologous to the virulence-associated type-III secretion systems (v-T3SS) of the injectisome devices (Abby and Rocha, 2012). Enteropathogenic *yersiniae* and *S. enterica* serovar Typhimurium produce flagella during colonization of the intestinal mucosa, but repress flagella production after invasion of the intestinal epithelial layer. The structural subunits, forming the flagellar filament, are critical because they are potent inducers of the innate immune system of the host (Young et al., 2000; Hayashi et al., 2001). Regulation of flagella production is tightly linked with the expression of the virulence-associated injectisome devices (v-T3SS) employed by many Gram-negative pathogens to manipulate host cells (Iyoda et al., 2001; Horne and Pruss, 2006; Mouslim and Hughes, 2014) (see also Attachment and Invasion of the Intestinal Epithelium).

The correct spatiotemporal assembly of the flagellum is a complex process that involves temporal regulation of more than 60 genes organized into a transcriptional hierarchy of three promoter classes (Kapatral et al., 2004; Chevance and Hughes, 2008; Anderson et al., 2010). The first levels of this hierarchy are similar between *Yersinia* and *Salmonella*. On top of the transcriptional regulatory cascade, many environmental stimuli are integrated at the level of the flagellar master regulatory operon, *flhDC*, which is expressed from a σ^70^-dependent flagellar Class 1 promoter (Yanagihara et al., 1999; Young et al., 1999; Kapatral et al., 2004). The active FlhD_4_C_2_ heteromultimeric complex (FlhDC) directs σ^70^-RNA polymerase (RNAP) to transcribe from flagellar Class 2 promoters (Liu and Matsumura, 1994; Wang et al., 2006), which regulate (i) the expression of the flagellar hook basal body (HBB) complex including the f-T3SS components needed for the export of all subsequent extra-cytoplasmic subunits and (ii) two regulators, the flagella-specific alternative sigma factor, σ^28^ (encoded by *fliA*) and its cognate anti-sigma factor, FlgM (Iriarte et al., 1995; Liu and Matsumura, 1995; Ding et al., 2009). Transcription from flagellar Class 3 promoters is specific for σ^28^-RNAP and occurs only after a functional HBB structure has been assembled (Karlinsey et al., 2000). The binding of FlgM to σ^28^ prevents premature σ^28^-RNAP-dependent Class 3 transcription and thereby mediates a temporal sensing of the assembly state of the flagellum (Ohnishi et al., 1992; Chadsey et al., 1998). Prior to completion of the HBB structure, the f-T3SS exports only early secretion substrates and does not recognize the late secretion substrate FlgM. Upon HBB completion, the f-T3SS undergoes a switch in secretion specificity and FlgM is recognized as a late secretion substrate and secreted from the cell, thereby allowing for σ^28^-dependent transcription from Class 3 promoters (Hughes et al., 1993; Kutsukake, 1994). Gene products transcribed from Class 3 promoters include structural components of the chemotaxis machinery, the filament, and motor-force generators. Overall the hierarchy of flagellar expression is similar in *Yersinia*. However, several differences include (i) the presence of three tandem flagellin genes (*fleA, fleB, fleC*) in *Y. enterocolitica*, (ii) a different set of flagellar genes that are controlled directly by FlhDC and FliA (Class IIIa), and (iii) different expression levels/operon structure of certain flagellar genes, e.g., *fliA* and *fliZ* (Horne and Pruss, 2006). Moreover, although a direct interaction of the *Y. pseudotuberculosis* FlgM protein to σ^28^ was confirmed and shown to repress expression of the single flagellin gene (*fliC*) in *Y. pseudotuberculosis*, a *flgM* mutant of *Y. enterocolitica* and *Y. pseudotuberculosis* is non-motile. This suggested that the flagellin genes might be post-transcriptionally regulated (Kapatral et al., 1996; Ding et al., 2009). In addition, other differences exist in the regulatory hierarchies driven by FliA and FlgM when compared to *Salmonella*, and additional sigma factors apart from σ^28^ (e.g., σ^54^) were found to be involved in the control of flagellar biosynthesis (Ding et al., 2009).

Expression of the flagella is tightly regulated and several layers of autoregulation exist that fine-tune expression of the flagellar system. The regulatory circuits are very well characterized in *Salmonella*. One regulatory circuit involves autoregulation of *flhDC* gene transcription. After activation of flagellar synthesis, the FlhDC complex initiates a regulatory feedback loop by activating transcription of its own repressor, RflM (Singer et al., 2013), which provides a mechanism to fine-tune *flhDC* expression levels. In *Salmonella*, the Class 2 gene product FliT also has been shown to have a dual function as an f-T3SS secretion chaperone of the filament cap protein FliD and as a regulator of FlhDC activity (Fraser et al., 1999; Yamamoto and Kutsukake, 2006). FliT negatively regulates FlhDC activity by inhibiting promoter binding of the FlhDC complex and increases ClpXP-dependent proteolysis of the FlhC subunit (Yamamoto and Kutsukake, 2006; Sato et al., 2014). Secretion of its cognate secretion substrate FliD after HBB completion releases FliT and thereby provides a mechanism to fine-tune flagellar numbers in response to the growth rate (Aldridge et al., 2010). Although the organization of the flagellar genes in operons and the arrangement along the chromosome differs between *Salmonella* and enteropathogenic *yersiniae*, the order and sequence of the flagellar genes, including *fliT* and *fliD* is mostly conserved (Horne and Pruss, 2006; Ding et al., 2009), suggesting that the overall functions might be maintained.

Another Class 2 gene product, FliZ, is a positive regulator of flagellar Class 2 and Class 3 gene transcription, which increases FlhDC activity by two distinct mechanisms (Kutsukake et al., 1999) (**Figure 2**). First, FliZ is a transcriptional repressor of the EAL-domain protein YdiV (Wada et al., 2011b). The YdiV protein binds to the FlhD subunit and prevents binding of FlhDC to Class 2 promoters by targeting FlhDC complex to ClpXP-mediated proteolytic degradation (Wozniak et al., 2009; Takaya et al., 2012). Expression of YdiV is high under nutrient starvation in *Salmonella* and low under high nutrient conditions (Wada et al., 2011a). Accordingly, *Salmonella* is only motile when nutrients are plentiful in contrast to many other bacteria (Wada et al., 2012). The second mechanism by which FliZ activates Class 2 and 3 gene transcription in *Salmonella* is via positive feed-forward regulation of invasion gene expression encoded on Spi-1 (Lucas et al., 2000; Iyoda et al., 2001). FliZ is needed for efficient induction of the Spi-1 system via post-translational activation of the Spi-1 regulatory protein HilD, which in turn activates *flhDC* gene transcription (Iyoda et al., 2001; Chubiz et al., 2010; Mouslim and Hughes, 2014; Singer et al., 2014).

Although much less is known about the regulatory components implicated in the control of *Yersinia* motility and flagella formation, a similar close regulatory cross-circuit between the flagellar (f-T3SS) and *Yersinia* virulence plasmid (pYV)-encoded v-T3SS/injectisome regulons was detected (**Figure 3**). However, the components implicated in the cross-talk appear to differ significantly. An upregulation of the *Yersinia* v-T3SS/*yop* regulon was observed in an *flhDC* mutant of *Y. enterocolitica* (Bleves et al., 2002). Several groups further reported that certain injectisome genes contain a consensus FliA promoter sequence, and Horne et al. observed a negative effect of the flagellar-specific alternative sigma factor σ^28^ on the expression of four injectisome genes (Iriarte et al., 1995; Kapatral et al., 1996; Horne and Pruss, 2006). This regulation seems to be indirect and mediated by the transcriptional master activator LcrF/VirF, which activates transcription of the majority of all v-T3SS and associated *yop* effector genes (Horne and Pruss, 2006). In addition, FliA was found to induce expression of the primary invasin gene *invA*, which is encoded between two flagellar operons (Badger and Miller, 1998; Horne and Pruss, 2006). Vice versa, this coregulation ensures coexpression of flagellar-mediated motility and invasin-promoted host cell invasion by enteric *yersiniae*.

As flagellar synthesis consumes a significant proportion of the cell’s biosynthetic capacities a plethora of environmental signals and a myriad of negative and positive control mechanisms are integrated on the level of *flhDC* gene expression, translation and FlhDC complex stability that ultimately decide the commencement of flagellar biosynthesis. The *flhDC* promoter of *Salmonella* displays a complex structure and multiple transcriptional start sites have been identified in recent transcriptome studies (Kröger et al., 2012, 2013). Transcriptional activity of the different promoters in *Salmonella* depends on the presence of different transcriptional activators such as cAMP-Crp, Fur, Fis (P1 promoter), HilD (P5 promoter), and the quorum sensing two-component regulatory system QseBC (Komeda et al., 1976; Yanagihara et al., 1999; Campoy et al., 2002; Kelly et al., 2004; Bearson et al., 2010; Mouslim and Hughes, 2014; Singer et al., 2014). Moreover, *flhDC* gene expression is also under negative control of the phosphorelay system RcsCDB, the LysR-family protein RflM/EcnR, the Spi-1-encoded regulator RtsB, the LysR-type regulator LrhA (RovM in *Yersinia*), and SlyA (RovA in *Yersinia*) with the P1 and P5 promoters being the main regulatory targets (Yanagihara et al., 1999; Wang et al., 2007; Singer et al., 2013; Mouslim and Hughes, 2014). The majority of these regulators is subjected to growth phase control and exerts its control at different stages during the bacterial growth phase. This dynamic separates the transcriptional activation of *flhDC* into a role for flagellar production during early growth phase from a role in virulence gene expression at later growth phase (Mouslim and Hughes, 2014). Finally, fimbriae-associated genes were also implicated in negative regulation of the *flhDC* operon. The *pefI* and *srgD* genes are encoded on the *Salmonella* virulence plasmid as part of the *p*lasmid-*e*ncoded *f*imbriae (*pef*) locus and function together in repression of *flhDC* transcription (Wozniak et al., 2009). FimZ is an activator of type 1 fimbriae and was shown to represses *flhDC*, as well as Spi-1 genes, which indicates that cross-talk between the flagellar, fimbriae and Spi-1 regulatory systems is of importance during the transition from a motile, planktonic lifestyle to intestinal colonization and persistence (Saini et al., 2010).

Much less is known about the regulation of *flhDC* expression in *Yersinia*, but varying expression levels under different environmental conditions (Nuss et al., 2014) suggest a similar complex regulation pattern. In fact, several transcriptional regulators are already known to influence flagella formation and motility. Analysis of *flhDC* expression in *Y. enterocolitica* revealed a role of OmpR/EnvZ in the positive control of the flagellar operon, which is in contrast to the negative role observed in *E. coli* (Hu et al., 2009). Furthermore, the two hierarchical quorum sensing LuxRI orthologs (YpsRI and YtbRI) of *Yersinia* control swimming motility via regulation of *flhDC* and *fliA*, whereby *N*-acylhomoserine lactones (AHLs) synthesized via YtbI activate *flhDC*, in conjunction with YpsR, but repress *fliA* in conjunction with YtbR and YpsR. A mutant analysis further indicated that quorum sensing regulates motility both positively (via YtbRI) and negatively (via YpsRI) (Atkinson et al., 1999, 2008). A recent study addressing gene regulation by the quorum sensing proteins YenR and YenI of *Y. enterocolitica* further demonstrated that YenR represses expression of *yenS*, encoding two non-translated RNAs 169 and 105 nucleotides long that share the same 5′ end, which play a stimulatory role in swarming motility (Tsai and Winans, 2011). Similar to *Salmonella*, the LrhA homolog RovM of *Yersinia* was found to repress motility by reducing the number of flagella in *Y. pseudotuberculosis*, whereas the RovM-dependent SlyA homolog RovA had no influence (Heroven and Dersch, 2006).

On the post-transcriptional level, it has been shown that also the carbon storage regulator CsrA is required for motility in both enteropathogenic *Yersinia* species (Heroven et al., 2008). For *Y. pseudotuberculosis* it was found that CsrA interacts directly with the 5′-untranslated region of the *flhDC* mRNA and stimulated FlhDC synthesis most likely by protecting the mRNA from RNase E cleavage similar to what has been observed in *E. coli* (Heroven et al., 2008). Recently, it has also been shown that CsrA is involved in the activation of the FlhDC/FliA cascade in *Salmonella*. Regulation of *flhDC* expression via CsrA is consistent with reports that the BarA/SirA TCS represses the flagellar regulon indirectly, most likely by regulating *csrB*, which in turn is an antagonist of CsrA activity (Teplitski et al., 2003, 2006). In *Salmonella*, CsrA is known to control the expression of the specific phosphodiesterase YhjH (STM3611), governing the synthesis of (3′-5′)-cyclic-diguanosine monophosphate (c-di-GMP), which reciprocally regulates flagella function and production of biofilm matrix components (Jonas et al., 2010). Furthermore, it has been reported that the BarA/SirA TCS represses the flagellar regulon indirectly, most likely by regulating *csrB* (Teplitski et al., 2003, 2006).

Temperature is a key environmental cue for *yersiniae*, and unlike *S. enterica* serovar Typhimurium, both enteropathogenic *Yersinia* species are not motile at 37°C. Loss of motility at body temperature is due to a rapid repression of the flagellar operons (Kapatral et al., 1996). Temperature-dependent regulation of the flagellar genes appears to occur through σ^28^/FliA, as a rapid reduction of *fliA* mRNA was observed at a temperature-upshift, whereas the *flhDC* master operon is transcribed in a temperature-independent manner (Kapatral et al., 2004; Nuss et al., 2015). Notably, in a *fliA*-deficient mutant, also the temperature-dependent expression levels of all plasmid-encoded v-T3SS/*yop* genes were considerably reduced (Horne and Pruss, 2006). This indicated that FliA further contributes to temperature regulation of crucial virulence genes, most likely through is influence on *lcrF/virF*.

### Attachment and Invasion of the Intestinal Epithelium

Enteric pathogens such as *salmonellae* and *yersiniae* are armed with a set of classical virulence factors (e.g., adhesins, invasins) that promote tight attachment of the bacteria to the mucosal surface of the intestinal tract and induce their passage through the intestinal epithelial layer into underlying lymphatic tissues. During the early stages of the infection both enteropathogenic *Yersinia* species as well as *S. enterica* serovar Typhimurium bind to and invade into M cells of the epithelium, overlaying the Peyer’s patches in the most distal part of the ileum (Grutzkau et al., 1990; Jones et al., 1994). The surface-exposed outer membrane protein invasin is the most efficient adhesion and internalization factor of enteropathogenic *yersiniae* (Marra and Isberg, 1997; Grassl et al., 2003; Handley et al., 2005). The invasin protein, which is required for the colonization of the intestinal tract of mice and pigs (Schaake et al., 2013), is in most enteric *Yersinia* strains predominantly produced at moderate temperature during stationary phase mimicking the free-living or food-associated lifestyle (Isberg et al., 1988; Pepe et al., 1994). This expression pattern seems to guarantee rapid internalization into M cells shortly after ingestion.

The MarR-type regulator RovA, a dimeric winged-helix DNA-binding protein of the SlyA/Hor/Rap family activates *invA* transcription in response to temperature (Revell and Miller, 2000; Nagel et al., 2001; Heroven et al., 2004). A detailed structure-functional analysis of RovA revealed that RovA is an intrinsic temperature-sensing protein – a protein thermometer – in which thermally induced conformational changes of a small loop in the dimerization domain (i) interfere with the DNA-binding capacity of the regulator, and (ii) render the regulatory protein more susceptible to proteolytic degradation by the Lon protease (Herbst et al., 2009; Quade et al., 2012; Uliczka and Dersch, 2012). An analysis comparing the invasion properties of *Y. enterocolitica* O:3 and O:8 strains revealed that RovA stability is enhanced in *Y. enterocolitica* O:3 strains due to a P98S substitution in RovA. *Y. enterocolitica* O:3 is an emerging pathogen using pigs with a higher body temperature (39–41°C) as preferred reservoir hosts, and this stabilized RovA variant was found to improve persistence of the pathogen in the porcine intestinal tract (Schaake et al., 2014; Valentin-Weigand et al., 2014). Most interestingly, SlyA, the close homolog of RovA from *Salmonella* with a very similar structure, is not a thermosensor and remains fully active and stable at 37°C. Introduction of only three amino acid substitutions, reflecting evolutionary replacements in SlyA, were sufficient to eliminate the thermosensing properties of RovA and prevent degradation (Quade et al., 2012). This indicated that only minor changes can transform a thermotolerant regulator into a thermosensor that allows adjustment of virulence and fitness determinants in response to the temperature of their environment. Strikingly, in contrast to RovA, SlyA is not involved in invasion or the colonization of the small intestine, but it is required for the survival and replication in host phagocytes and destruction of M cells during later stages of the infection (Libby et al., 1994; Daniels et al., 1996; Buchmeier et al., 1997; Watson et al., 1999; see Regulatory Circuits Controlling Later Stages of Infection and Defense Systems against the Host’s Immune Response).

Transcription of *rovA* and the RovA-regulated *invA* gene is also subjected to silencing by the ancestral nucleoid-structuring protein H-NS (Heroven et al., 2004; Ellison and Miller, 2006) (**Figure 3**), a global regulator implicated in the xenogeneic repression of many virulence and physiological genes acquired by horizontal gene transfer (Navarre et al., 2006; Dorman, 2007). H-NS and RovA bind preferentially to AT-rich regions and their binding sites are superimposed in both the *invA* and *rovA* regulatory region (Heroven et al., 2004). Active RovA alleviates transcriptional repression by H-NS and stimulates the activity of the RNAP (Tran et al., 2005). This anti-silencing mechanism seems common in *Yersinia*, as several other genes have been identified, that are controlled by both regulators (Cathelyn et al., 2007). It is also observed in many other *Enterobacteriaceae*, including *Salmonella*, where H-NS promotes silencing of invasion gene regulators encoded on Spi-1, apparently to buffer fitness costs that were associated with acquisition and expression of host colonization factors (Olekhnovich and Kadner, 2007).

H-NS is also capable to form heterodimers with members of the Hha/YmoA family. Hha and YmoA are small basic proteins that have been shown to participate in the modulation of virulence gene regulation in different Gram-negative bacteria (Madrid et al., 2006; Stoebel et al., 2008). Formation of YmoA-H-NS complexes has been shown to modulate expression of invasion and several other H-NS-dependent virulence genes in *Yersinia* (Ellison et al., 2003; Heroven et al., 2007) and in *Salmonella*. However, the molecular mechanism how YmoA/Hha modulates H-NS is not well understood since its influence varies considerably among the H-NS-dependent genes in the different microorganisms. Nonetheless, it is evident that the global influence of H-NS enables the pathogens to link this set of virulence-relevant genes with other environmental control systems. For instance, the H-NS modulator YmoA is preferentially degraded at 37°C by the ClpP and Lon proteases in *Yersinia* (Jackson et al., 2004), and in *Salmonella* it has been shown that transcription of the *hn*s gene – which influences expression of the Spi-1-encoded invasion genes through the regulator HilD – is repressed by the iron homeostasis regulator Fur (Troxell et al., 2011). Metal-bound Fur has also been shown to bind to an AT-rich region of the *hilD* promoter to stimulate *hilD* transcription (Ellermeier and Slauch, 2008; Teixido et al., 2011).

The LysR-type regulator RovM (LrhA in *Salmonella*) was identified as another repressor of *rovA* expression, and full repression of *rovA* expression can only occur through the cooperation of RovM with H-NS (Heroven and Dersch, 2006). RovM synthesis is only activated under nutrient limiting conditions, and this strongly suggested that dependency of *rovA* and *invA* expression on the availability of nutrients is mainly mediated through RovM (Heroven et al., 2007). In fact, later attempts to unravel the molecular regulatory mechanism of *rovM* revealed that the nutrient and ion-controlled Csr system is responsible for *rovM* expression in response to the supply of carbon sources of the growth media (Heroven et al., 2008) (**Figure 3**). Overexpression of UvrY, CsrB, and CsrC resulted in a strong decrease of RovM levels and an increase of *rovA* transcription, and high amounts of the RovA protein can be detected in a *csrA* mutant in minimal media in which *rovA* expression is normally fully repressed (Heroven et al., 2008; Bücker et al., 2014). A detailed expression analysis further revealed that in contrast to the *Salmonella* Csr system, both CsrB and CsrC are counter-regulated and respond to different TCS in response to metabolites (UvrY/BarA-CsrB) or ions (PhoP/PhoQ-CsrC) and are oppositely controlled by the Crp protein (Heroven et al., 2008; Nuss et al., 2014). Apart of its influence on the RovM-RovA-InvA regulatory cascade, the *Yersinia* Csr system and Crp are part of a large adaptive response network, adjusting metabolic, and physiological processes, stress adaptation and virulence gene expression in response to changing environmental conditions (Heroven et al., 2012a; LeGrand et al., 2015; Nuss et al., 2015; Vakulskas et al., 2015). A recent study elucidated a tight connection between pathogenicity and core metabolism by integrated transcriptome and ^13^C-fluxome analysis of *Y. pseudotuberculosis*, and identified the pyruvate-TCA cycle node as a focal point for controlling the host colonization and virulence of *Yersinia* which is tightly controlled by the interplay of Crp and the Csr system (Bücker et al., 2014).

In contrast to *Yersinia*, initial invasion and transcytosis of *Salmonella* through the intestinal layer requires the expression of the Spi-1-encoded injectisome system, which is controlled by a very distinct regulatory cascade (**Figure 2**). The appropriate environmental conditions to allow invasion are sensed by a myriad of regulators encoded both inside and outside of the pathogenicity island (PAI). A key regulator of Spi-1 is the OmpR/ToxR family protein HilA, and all environmental signals that sense optimal conditions for Spi-1 expression are integrated on the level of HilA expression (Bajaj et al., 1996; Ellermeier and Slauch, 2007). HilA is a positive regulator that primarily activates transcription of genes encoding structural components of the Spi-1 injectisome system, including the *prg/org* and *inv/spa* operons (Lee et al., 1992; Bajaj et al., 1995) and other virulence genes outside Spi-1, including the Spi-4 encoded *sii* operon (Gerlach et al., 2007). *hilA* gene transcription is controlled by a complex feed-forward loop comprised of the three AraC-like regulatory proteins HilC, HilD, and RtsA encoded on Spi-1 or on a separate 15 kb island (Schechter and Lee, 2001; Ellermeier and Slauch, 2003; Ellermeier et al., 2005) (**Figure 2**). Each of the three regulators can independently bind and activate the *hilA* promoter, their own gene, and the genes of the respective other regulators by counteracting silencing mediated by H-NS and Hha (YmoA in *Yersinia*) (Olekhnovich and Kadner, 2002; Ellermeier et al., 2005; Olekhnovich and Kadner, 2006). It has been proposed that the feed-forward loop system of HilC, HilD, and RtsA functions as a switch that controls *hilA* expression by modulating the threshold of HilD protein required for the ultimate HilA activation. In this context, HilD is the key activator of *hilA* transcription and various environmental signals are known to influence HilD production and activity, whereby RtsA and HilC function as amplifiers of the activating signal (Ellermeier and Slauch, 2007). Moreover, HilD coordinates *hilA* transcription with the expression of other crucial infection-relevant systems, e.g., it induces expression of the flagellar operons through activation of *flhDC* transcription and controls expression of Spi-2 genes (see also the Sections Regulation of Motility and Regulatory Circuits Controlling Later Stages of Infection and Defense Systems against the Host’s Immune Response).

The temporal coordination of flagellar and Spi-1 gene expression plays an important role during the initial phase of the *Salmonella* infection cycle when the bacteria are initially motile in the lumen and subsequently turn on Spi-1 virulence genes needed for invasion of the epithelial layer. This is achieved by the flagellar-encoded regulator FliZ, which simultaneously represses type 1 fimbriae and activates Spi-1 via HilD (Iyoda et al., 2001; Chubiz et al., 2010). In the following stages flagellar gene expression is downregulated on the level of the *flhDC* promoter by the combined action of multiple regulators, including the Spi-1-dependent RtsB protein and the type 1 fimbriae regulator FimZ (Ellermeier and Slauch, 2003; Saini et al., 2010; Mouslim and Hughes, 2014; Singer et al., 2014). At later stages of the infection, synthesis of Spi-1 stops and expression type 1 fimbriae is upregulated (Saini et al., 2010). It is presumed that the expression of adhesive structures is needed for colonization and persistence of bacteria that were unable to breach the intestinal epithelium during the initial infection (Saini et al., 2010).

Based on the importance of HilA and HilD for Spi-1 gene expression, it is not surprising that various regulatory factors control their synthesis and activity in response to environmental conditions and ion/nutrient availability (**Figure 2**). Among them are the *Salmonella*-specific negative regulator HilE, which interacts with HilD and affects its activity, and multiple conserved regulatory factors: (i) the acyl-CoA synthetase FadD, (ii) the EnvZ/OmpR, PhoP/PhoQ and the Rcs phosphorelay systems controlling *hilD* or *hilA* transcription (Bajaj et al., 1995; Pegues et al., 1995; Garcia-Calderon et al., 2007), (iii) the DNA adenine methylase Dam and the degradosome (RNaseE), which regulates *hilA* and/or *hilD* mRNA stability and translation (Fahlen et al., 2000; Boddicker et al., 2003; Lopez-Garrido et al., 2014), (iv) the Lon and ClpXP protease (Kage et al., 2008), and (v) the BarA/SirA-CsrABC signal cascade in which CsrA negatively affects *hilD* translation by binding the ribosome-binding site of the *hilD* mRNA (Baxter et al., 2003; Ellermeier et al., 2005; Chubiz et al., 2010; Martinez et al., 2011). Apart from H-NS and Hha the *f*actor for *i*nversion *s*timulation (Fis), also controls Spi-1 gene expression. Fis-mediated activation of the Spi-1 genes occurs through different regulatory modes: Fis binds to promoters of Spi-1 encoded genes, or controls conserved upstream regulators such as OmpR to influence the Spi-1 regulator HilD. Fis binds preferentially to supercoiled DNA, and Fis-promoted activation coincides with a high level of nucleoid supercoiling under anaerobic conditions in the gut during the initial infection of *Salmonella* (Wilson et al., 2001; Wang et al., 2013).

### Regulatory Circuits Controlling Later Stages of Infection and Defense Systems against the Host’s Immune Response

Efficient dissemination and long-term persistence of *S. enterica* serovar Typhimurium and both enteric *Yersinia* species in deeper tissues require the expression of an additional set of virulence genes, which allow them to resist host immune responses. The most important defense system of *yersiniae* is encoded on the virulence plasmid pYV. It includes the *yadA* adhesin gene, the *ysc* genes encoding a v-T3SS/injectisome and its dedicated *yop* effector genes (Cornelis et al., 1998), which are important to survive and multiply in the lymphoid tissues of their host. This integrated defense system prevents uptake and elimination of the extracellular pathogens by professional phagocytes. It disarms their phagocytic function, disrupts their communications, and induces their apoptosis by the injection of the Yop effector proteins which interfere with the cytoskeletal structure and certain signal transduction pathways (Bliska et al., 2013). All these virulence genes belong to the same regulon and their transcription is activated by the AraC-type regulator LcrF(VirF) in response to temperature and host cell contact (Bolin et al., 1988; Pettersson et al., 1996). A subset of the genes is also regulated by the presence of Ca^2+^, a phenomenon, which is referred to as *l*ow *c*alcium *r*esponse (LCR) (Goguen et al., 1984; Straley et al., 1993). *ysc/yop* genes were also found to be regulated by proteins that antagonize LcrF(VirF). One example is YtxR which competes with LcrF(VirF) for binding to the *yopE* and *yopH* promoters (Axler-Diperte et al., 2006). Another regulator is LcrQ (YscM1 and YscM2 in *Y. enterocolitica*), a factor, which is implicated in a feedback circuit that represses *ysc-yop* gene expression when Yop secretion is inhibited, e.g., in the absence of host cell contact. LcrQ(YscM1/YscM2) in cooperation with the YopD-LcrH complex interacts with 5′-UTRs of multiple *ysc/yop* mRNAs to block translation. Upon host cell contact LcrQ(YscM1/YscM2) is secreted by the Ysc/Yop v-T3SS machinery, repression is relieved due to lower LcrQ concentration in the cytoplasm and results in the upregulation of the *yop* and *ysc* genes (Stainier et al., 1997; Anderson et al., 2002; Chen and Anderson, 2011; Kopaskie et al., 2013).

Thermal control of the Ysc/Yop regulon is mediated through regulation of *lcrF*(*virF*) and this involves repression of LcrF(VirF) synthesis by the thermosensitive modulator YmoA and an RNA thermometer. The YmoA-H-NS complex was shown to repress *lcrF* transcription at moderate temperatures, but this repression is eliminated at 37°C due to rapid degradation of the YmoA protein by the Lon and ClpP proteases (Jackson et al., 2004; Heroven et al., 2007; Böhme et al., 2012). Most likely, this derepression is supported by thermally induced changes of the pYV DNA topology (Cornelis et al., 1989; Michiels et al., 1991; Rohde et al., 1994, 1999). Furthermore, it has been demonstrated that the 5′-UTR of the *lcrF*(*virF*) mRNA forms a temperature-sensitive two-stem-loop structure (RNA thermometer) at moderate temperatures in which the Shine-Dalgarno sequence is sequestered in the stem of the second hairpin and prevents *lcrF* mRNA translation (Hoe and Goguen, 1993; Böhme et al., 2012). However, an upshift to 37°C within the host leads to partial denaturation, and opening of the structure allows efficient ribosome binding and *lcrF* mRNA translation (Böhme et al., 2012). Besides temperature, LcrF(VirF) is also affected by other environmental signals. A genetic screen led to the identification of the transcriptional regulator IscR, which modulates gene transcription depending on the coordination of its 2Fe–2S clusters which can be influenced by oxidative stress, oxygen limitation, or iron availability (Miller et al., 2014). Similar to the *Salmonella* v-T3SS regulator HilD, LcrF expression seems also under control of several conserved TCSs: (i) the Rcs phosphorelay which is used to adapt their cell physiology in response to perturbations of the cell envelope (Li et al., 2015), (ii) the BarA/UvrY(SirA) controlling the Csr system in response to carbon sources (LeGrand et al., 2015), and (iii) the CpxAR system that responds to extra-cytoplasmatic stress (Liu et al., 2012). As mentioned above (see Regulation of Motility), LcrF synthesis is also under negative control of the flagellar sigma factor FliA/σ^28^, and this inverse regulation of flagellar and v-T3SS genes ensures that expression of the immune defense apparatus is repressed when *Yersinia* uses flagellar motility to colonize external habits at temperatures below 30°C (Horne and Pruss, 2006).

In contrast to *yersiniae*, which persist and replicate predominantly extracellularly in lymphatic tissues, *salmonellae* can actively invade, survive and proliferate efficiently within so-called *Salmonella-c*ontaining *v*acuoles (SCVs) inside the eukaryotic cytoplasm (**Figure 1**). The establishment and integrity of the SCV membrane is ensured by the action of effectors translocated via another horizontally acquired injectisome device encoded on Spi-2. Expression of Spi-2 and downregulation of Spi-1 gene expression marks an important transition from the invasion mode to the intracellular survival stage that is crucially dependent on the correct spatiotemporal action of a variety of activators and repressors of gene expression. The main regulatory proteins involved in the expression of Spi-2 include the two-component systems SpiR(SsrA)/SsrB, PhoP/PhoQ, OmpR/EnvZ, as well as the DNA-binding proteins HilD, SlyA (RovA in *Yersinia*), RcsB, the iron regulator Fur, and the nucleoid-associated proteins H-NS, Hha (YmoA in *Yersinia*), YdgT, IHF, and Fis (Fass and Groisman, 2009; Choi et al., 2014). The membrane-embedded *Salmonella*-specific sensor kinase SpiR(SsrA) and the response regulator SsrB are the primary activators essential for Spi-2 expression (Walthers et al., 2007), which is induced within the acidic environment of the SCV (Miao et al., 2002; Mulder et al., 2015). SsrB binds to promoter regions of all Spi-2-encoded gene clusters and counteracts H-NS-mediated silencing (Walthers et al., 2007). SsrB was also recently shown to downregulate the production of flagellar components in SCVs in macrophages (Brown et al., 2014), which would diminish flagellin-dependent stimulation of the NLRC4 inflammasome (Franchi et al., 2006; Miao et al., 2006). In addition to SsrB, also the SlyA protein (an ortholog of RovA of *Yersinia*) is implicated in the activation of several Spi-2 genes, mostly to overcome H-NS-mediated silencing (Okada et al., 2007). In contrast to RovA, SlyA does not function as a thermosensor and acts as a negative regulator of flagellar genes at 37°C via repression of *flhDC* (Quade et al., 2012; Mouslim and Hughes, 2014). In addition, two other homologous nucleoid-associated proteins, YdgT and Hha (YmoA in *Yersinia*) which form heterocomplexes with H-NS prevent Spi-2 gene expression and appear to be important in particular when the bacteria are extracellular (Coombes et al., 2005; Silphaduang et al., 2007).

Similar to the injectisome machinery of *Yersinia*, expression of the Spi-2 encoded injectisome genes of *Salmonella* seems to depend on the DNA topology and nucleoid-associated factors that modulate the transcription of the genes by the introduction of conformational changes. One of the modulators, the global DNA-bending protein *i*ntegration *h*ost *f*actor (IHF) is required for the expression of motility, Spi-1 and Spi-2 genes during the transition from exponential to stationary growth (Mangan et al., 2006). Also Fis, which also promotes Spi-1 gene expression (see Attachment and Invasion of the Intestinal Epithelium), is needed for full expression of Spi-2. Fis binds directly to the promoter regions of *spiR* and *ssaG* (Kelly et al., 2004; Lim et al., 2006) and its expression correlates with Spi-2 gene expression inside macrophages (O Croinin et al., 2006). Oxidative stress sensed during later intracellular infection stages results in relaxation of the nucleoid, which activates expression from Spi-2 promoters. Fis seem to accelerate relaxation and stabilizes the promoter topology of *ssrAB* to allow timely expression of Spi-2 genes (O Croinin et al., 2006; Dillon and Dorman, 2010).

Spi-2 is also controlled by several TCSs that are also implicated in the control of the colonization and invasion factors. Spi-2 genes are activated by the PhoP/PhoQ system that is essential for virulence and survival within macrophages (Miller et al., 1989). The response regulator PhoP controls Spi-2 by binding to the *ssrB* promoter region and the 5′-UTR of the *spiR* transcript (Bijlsma and Groisman, 2005). The OmpR/EnvZ two-component system also functions as an activator of Spi-2 by direct binding to both promoter regions of the *spiR*/*ssrB* system (Lee et al., 2000; Feng et al., 2003). The phosphorelay system RcsCDB has been shown to have a dual regulatory role as repressor of flagellar (*flhDC*) gene expression and activator of Spi-2 expression (Wang et al., 2007).

Expression of the Spi-2 genes is further tightly coordinated with expression of the Spi-1 genes. Cross-talk between the different PAIs is mainly mediated by the Spi-1-encoded regulator protein HilD. HilD antagonizes silencing via H-NS of both, the *hilA* and *spiR* promoters, but significantly higher levels of HilD protein are required for *spiR* promoter binding. This may explain why HilD regulates Spi-1 and Spi-2 differentially (Bustamante et al., 2008). While the physiological role of the HilD-mediated cross-talk between Spi-1 and Spi-2 remains to be further analyzed *in vivo* inside host cells, it has been proposed that coordinated regulation of Spi-1 and Spi-2 gene expression involves also IHF (as described above) which influences HilD expression (Fass and Groisman, 2009).

## Conclusion and Outlook

Crucial to the *Yersinia* and *Salmonella*’s capability to cause a successful infection is their ability to coordinate the expression of a plethora of virulence genes with numerous metabolic and stress adaptation functions required for survival in the different tissues during the course of the infection. To optimize their biological fitness, which is essential to compete with the intestinal microbiota and defend detrimental host responses, they only synthesize pathogenicity factors when they are needed. The importance of the physiological control of virulence-relevant determinants is supported by the fact that their constitutive or deregulated expression can strongly attenuate virulence. In order to govern environmental changes, host stresses and competition, the pathogens generally employ signal transduction systems that sense and respond to particular environmental parameters and host signals by modifying the level and/or activity of transcriptional networks and post-transcriptional control systems including regulatory RNAs, modulator proteins and signal molecules to fine-tune the expression of pathogenicity factors.

Pathogenicity genes are often organized in clusters or large genomic islands on the chromosome or on plasmids which were acquired via horizontal gene transfer. The genetic linkage of the determinants on these so-called PAIs allows the bacteria to coordinately control a set of functionally related virulence factors within a complex regulatory network. Most frequently the virulence operons of the PAIs are under direct control of pathogen-specific transcriptional master regulators (mostly of the AraC family), which are encoded on the PAI (e.g., HilA, HilD in *Salmonella*, LcrF in *Yersinia*). In addition to expression of the PAI-encoded genes, also loci outside the PAI can be controlled by the PAI master regulators (e.g., HilD controls flagellar and Spi-2 gene expression). This superimposed control and cross-talk between the different virulence systems enables the pathogens to coordinate the dynamic and order of virulence gene expression in response to the conditions imposed on the pathogen by the colonized host niches. The environmental conditions include availability of carbon/energy sources, oxygen, growth phase, pH, and osmolarity and are transmitted to the regulatory network via ancestral global TCS (e.g., EnvZ/OmpR, PhoP/PhoQ, and BarA/UvrY(SirA). In addition, global regulatory proteins/systems such as Crp, CsrABC, SlyA(RovA), and LrhA(RovM), which adjust virulence gene expression with stress responses, physiological features (biofilm formation/motility) and crucial metabolic functions are included into this network. Another important conserved regulatory feature is the silencing of pathogenicity gene clusters by the nucleoid-associated protein H-NS due to its binding preference to DNA with a higher %AT content. H-NS promoted silencing prevents unwanted virulence gene expression under non-infection conditions (i.e., in environmental niches) to avoid fitness loss. This effect can be counter-balanced and modulated by other H-NS-like proteins such as Hha(YmoA) which modify H-NS promoted silencing by heterocomplex formation under certain growth conditions. Furthermore, other DNA-bending modulators such as IHF and Fis counteract H-NS repression and support virulence gene activation by the virulence cluster/PAI-specific activator proteins.

Many of the contributing TCSs, global regulators and regulatory systems, and gene silencer/modulator proteins are conserved between *Yersinia, Salmonella* and many other *Enterobacteriaceae*. However, their interactions, arrangement within the complex regulatory network and composition of the control elements vary between the related bacteria. In fact, small variations of the content and organization of the genetic information of the pathogens over time, e.g., point mutations, gene rearrangements, deletions, and insertion of foreign DNA can lead to rapid and fundamental changes. These genetic modifications are the primary forces, which bring out phenotypic differences leading to (i) distinct pathogenic properties and evolution of distinct species, and (ii) adaptation to different environments and alterations of their life-style. In fact, results from our previous work clearly demonstrated that even very small variations provoke major differences in the virulence properties of related pathogens. For instance, a 20 bp insertion can transfer the *Yersinia* adhesin YadA into an invasin (Heise and Dersch, 2006). Moreover, only three amino acid substitutions can switch the thermotolerant regulatory protein SlyA of *Salmonella* into a protein thermometer similar to the orthologous *Yersinia* regulator RovA (Quade et al., 2012). Variations found between the *Salmonella* Hha and the *Yersinia* YmoA protein render YmoA but not Hha susceptible to proteases at 37°C (Jackson et al., 2004), and a 2 nt exchange can modify a 5′-UTR into an RNA-thermometer (Steinmann and Dersch, 2013). These alterations of crucial control elements can switch thermotolerant gene regulatory circuits into a thermo-responsive control system, which allows pathogens such as *Yersinia* to inhabit a wide range of environmental, insect-vector- and mammalian host-associated niches.

Another benefit of the complex arrangement of the different regulatory factors is that some regulatory circuits enable heterogenous/bistable expression of certain virulence genes. As a result, a genetically identical bacterial population can consist of subpopulations that express (ON state) or do not express (OFF state) certain virulence factors. This so-called phenotypic heterogeneity can confer clonal subsets of pathogens with different virulence properties, metabolic functions, and/or physiological features. Recent work by Davis et al. (2015) has identified three subpopulations of *Y. pseudotuberculosis* within microcolonies at a single tissue site. The most peripherally localized bacteria which contact neutrophils express anti-phagocytic virulence factors, e.g., YopE, the bacteria growing on the exterior of the microcolony induce the nitric oxide (NO)-detoxifying gene *hmp* and prevents NO diffusion and elimination of the interior bacterial population and illustrates a sophisticated form of division of labor during infection (Davis et al., 2015). Another paradigmatic example of cooperative virulence is the bistable expression of the *Salmonella* Spi-1 injectisome and flagellar genes. This results in the generation of one subpopulation that expresses Spi-1 genes. A fraction of theses cells invade the intestinal epithelium and induce inflammation, whereas other motile subpopulations consume host products released during inflammation, This enables *Salmonella* to outcompete the intestinal microbiota and establish a productive infection in a cooperative manner (Ackermann et al., 2008; Diard et al., 2013). The invasive subpopulation is also characterized by a lower growth rate, which is associated with tolerance to certain stresses including the exposure to antibiotics (Diard et al., 2013). This property is referred to as bet-hedging; one subpopulation expresses features optimized for the present environment allowing it to survive and proliferate, whereas another part of the population expresses a phenotype less well adapted to the momentary niche, but adjusted to a state the environment might turn into. This behavior allows a population to survive in an unpredictable and frequently fluctuating environment, as experienced by the pathogen during the different stages of an infection. Future research addressing virulence gene expression in different tissues throughout the infection will elucidate whether phenotypic heterogeneity is restricted to special regulatory circuits or a more general control scheme. Existence of pathogen subsets cannot only hamper the control of infection by certain antibiotic therapies, but it can result in the failure of novel anti-virulence strategies directed against crucial virulence traits, e.g., T3SSs to combat bacterial diseases. An attractive alternative in this context would be the development of anti-virulence strategies directed against major global virulence regulators, which are essential for virulence as they coordinate multiple virulence traits with crucial metabolic and physiological functions of the pathogen.

## Conflict of Interest Statement

The authors declare that the research was conducted in the absence of any commercial or financial relationships that could be construed as a potential conflict of interest.
